# Mechanisms of Drug Desensitization: Not Only Mast Cells

**DOI:** 10.3389/fphar.2020.590991

**Published:** 2020-12-23

**Authors:** Alessandra Vultaggio, Andrea Matucci, Francesca Nencini, Susanna Bormioli, Emanuele Vivarelli, Enrico Maggi

**Affiliations:** ^1^ Immunoallergology Unit, Careggi University Hospital, Florence, Italy; ^2^ Immunology and Cellular Therapy, Careggi University Hospital, Florence, Italy; ^3^ Translational Immunology Unit, Immunology Area, Pediatric Hospital Bambino Gesù, IRCCS, Rome, Italy

**Keywords:** desensetisation, allergy (hypersensitive anaphylaxis), mast cells, T reg cell, IL-10, IL-35

## Abstract

Drug desensitization (DD) allows transient clinical tolerance to the drug in reactive patients and it is frequently and successfully used in the management of both IgE and non IgE-mediated hypersensitivity reactions (HRs). The underlying mechanisms behind this process is not well understood. The desensitization procedure is associated with the inhibition of mast cells degranulation and cytokine production, that, is attributable, at least partially, to the abrogation of Ca2+ mobilization; *in vitro* findings and *in vivo* mouse models of rapid desensitization show that the organization and spatial distribution of actin is critical for Ca2+ mobilization. Some clinical observations may suggest the induction of a longer memory of tolerance by DD and they raise the suspicion that other cells and mechanisms are involved in DD. Some data are emerging about the modifications of immune responses during DD in patients with previous immediate HRs. In particular, an increase of regulatory cytokines, mainly represented by IL-10, has been shown, and more importantly, the appearance of IL-35 producing T regulatory cells has been described during DD. The release of controlled cellular mediators by mast cells over time and the development of the antigen-specific regulation of adaptive response allow to safely and successfully reach the target dose of a first line drug during DD.

## Introduction

The treatment of many disorders including cancer and autoimmune diseases can be complicated by hypersensitivity reactions (HRs). Clinical manifestations vary considerably, ranging from mild to severe and life-threatening reactions leading to drug discontinuation, which in turn can decrease patients’ quality of life and/or life expectancy (Vultaggio et al., 2011; Sala-Cunill et al., 2019). Phenotypes in drug allergy focus on symptoms and timing, classifying the reactions as immediate or delayed, depending on the time between treatment administration and the onset of symptoms (Bonamichi-Santos and Castells, 2016). The most frequently involved culprit drugs are represented by antibiotics, aspirin, chemotherapeutics (mainly platinum compounds and taxanes) and biological agents (Vultaggio and Castells, 2014).

Management of HRs, beyond an allergological work-up aimed to define the pathogenic mechanism of the reaction, may include drug desensitization (DD) when there is no alternative therapy available. The culprit drug is usually avoided in order to prevent future reactions and DD was developed as a treatment option to maintain patients on first line therapy (Castells, 2017).

The activation of mast cells (MC) plays a critical role in HRs, not only limited to the immediate release of an array of preformed inflammatory mediators including histamine, tryptase, serotonin, chymases, cytokines, and growth factors, but also *de novo* synthesis of lipid mediators such as leukotrienes. In addition to the classical IgE-mediated MC activation, other mechanisms may be involved. Some medications can directly activate MC via the recently identified Mas-related G Protein Coupled Receptor-X2 (MRGPRX2) transmembrane protein, as in the case of fluoroquinolones, neuromuscular blocking agents, and vancomycin (McNeil et al., 2015; Boyce, 2019). Others may further activate mast cells via complement activation leading to the production of anaphylotoxins C3a and C5a (Jimenez-Rodriguez et al., 2018). The direct activation of membrane receptors as in the case of opioids and estrogens, represent a non-immunological pathway of MC activation possibly involved in the induction of HRs (Spoerl et al., 2017). Finally, cyclooxygenase-1 inhibition [as in the case of Aspirin Exacerbated Respiratory disease (AERD)] may occur.

The aim of this review is to evaluate the mechanisms involved in successuful DD, highlighting the role of regulatory cells and cytokines in the modulation of a drug-specific immune response.

## Drug Desensitization: General Concepts

Management of HRs in patients without treatment alternatives is based on the DD procedure, able to induce a temporary hyporesponsive state by incremental escalation of sub-optimal doses of the offending drug, until reaching required dosage (De las Vecillas Sanchez et al., 2017). Drug desensitization was developed due to the pressing need to reintroduce drugs in a safe fashion in patients who had developed both IgE-and/or non IgE-mediated HRs to critical drugs. Because DD is able to induce a temporary tolerance to the culprit drug, and considering that some medications (chemotherapy, biologic agents) have prolonged dosing intervals, subsequent administrations must be preceded by a DD procedure in order to overcome the loss of tolerance.

Desensitization is conceptually dedicated to patients in which an IgE-mediated mechanism is demonstrated by positive skin testing or serum IgE for culprit drug, however, patients who suffered immediate reactions to taxanes and other chemotherapies in which the IgE mechanisms cannot be demonstrated have also been successfully desensitized (Madrigal-Burgaleta et al., 2019).

Two types of DD protocols are available: rapid drug desensitization which addresses type I reactions with mast cells/basophils/IgE involvement, and slow drug desensitization which addresses delayed type IV reactions with T-cell involvement (Castells, 2015). Mixed reactions however have become more frequent, so DD protocols have slowly changed the segregation paradigm of DD vs. slow drug desensitization (Pyle et al., 2014). Desensitization is contraindicated in patients whose reaction suggests a history of severe cutaneous reactions, such as Stevens-Johnsons syndrome, toxic epidermal necrolysis, drug induced hypersensitivity syndrome, drug reaction (rash) with eosinophilia and systemic symptoms and acute generalized exanthematous pustulosis. Desensitization is also not considered appropriate for reactions of serum sickness or haemolytic anemia (Castells, 2017). Omalizumab is a humanized IgG1 monoclonal antibody, initially approved for the treatment of severe allergic asthma and more recently, for the treatment of chronic idiopathic urticaria. In several case reports it has been applied to control the reactions occurring during DD, for aspirin (Waldram et al., 2018), insulin (Mishra et al., 2018), elosulfasi α (Guvenir et al., 2017), carboplatin (Oude Elberink et al., 2020), and oxaliplatin (Prieto-Garcia et al., 2019).

## Mechanisms Involved In Drug Desensitization: Mast Cells

Regardless of whether the reaction is the consequence of an IgE- or non IgE-mediated mechanism, MC are key effector cells in the majority of immediate drug reactions, and the desensitization procedure is associated with the inhibition of MC degranulation and cytokine production.

Both *in vivo* and *in vitro* studies have been used to understand the cellular and molecular pathways influencing the function of MC and basophils during DD.

Several observations displayed negative skin testing after desensitization, indicating inhibition of the MC activation. These data have been extensively described in DD for chemotherapeutics and more recently for biological agents (Lee et al., 2004). By using sensitized bone marrow-derived MC under physiologic calcium conditions and by administrating incremental doses of the drug at fixed time intervals, cells were shown to become unresponsive (Sancho-Serra et al., 2011). Recent data obtained in a subject sensitized to infliximab (IFX) and grass pollen, who experienced an immediate HR to IFX, showed that skin testing for IFX was positive before cycles of DD but negativized after each procedure, while skin testing for grass pollen remained positive before and after each cycle (Vultaggio et al., 2020). These data obtained in humans were confirmed by *in vitro* results. In fact, challenging with the culprit drug after being desensitized did not induce *in vitro* activation of MC that could still be activated by different antigen stimulation, supporting the concept that DD is an antigen-specific process (Gladys et al., 2016).

To understand the mechanisms by which DD procedures impact MC mediator release, it is useful to define sequential events starting from IgE/FcεRI cross-linking to the intracellular signals.

Phosphorylation of subunit ITAMs (immune-receptor tyrosine-based activation motif) is important in initiating and inducing downstream propagation of intracellular signaling (Phong et al., 2015). Activated Lyn initiates signal transduction through phosphorylation of the β and γ ITAM chain. In the first phase of the process, phospholipase Cγ phosphorylates and then hydrolyzes phosphatidyl inositol bisphosphate to yield inositol trisphosphate (IP3) and diacylglycerol (DAG). IP3 induces an increase in cytosolic calcium ion (Ca2+) concentration, by binding to its receptor in the endoplasmic reticulum and rapidly inducing the process of calcium mobilization. In the subsequent phase, a prolonged Ca2+ influx occurs (Nam and Kim, 2020).

Although several studies have attempted to examine the underlying mechanisms regarding the effects of DD on MC, a general consensus is not yet reached in the literature.

Initial studies suggested that MCs became unresponsive after DD as the consequence of internalization of FcεRI through progressive cross-linking at low antigen concentrations (Shalit and Levi-Schaffer, 1995; Morales et al., 2005). More recent studies have shown that antigen/IgE/FcεRI may remain on the surface during DD (Gladys et al., 2016). In particular, it has been shown that surface IgE was not completely internalized, leaving enough IgE bound on the cell surface to bind Ag and potentially cause degranulation.

It would seem that MCs’ hypo-responsiveness is attributable, at least partially, to the abrogation of Ca2+ mobilization, a critical determinant of degranulation and cytokine production in mast cells (Gladys et al., 2016). Specifically, the organization and spatial distribution of actin is critical for Ca2+ mobilization in several cell types, including MCs, as demonstrated by *in vitro* findings and *in vivo* mouse models of rapid desensitization.

Of note, MRGPRX2-related MC degranulation are probably to DD preocedure as MRGPRX2 receptor does not undergo internalization.

## Other Mechanisms Involved In Drug Desensitization: Not Only Mast Cells

It is currently assumed that drug tolerance induced by DD is not a permanent state and that it is sustained by a pharmacologic, and not immunologic, tolerance. However, some clinical observations may suggest the induction of a longer memory of tolerance by DD.

Firstly, the rate of reactions during DD procedures progressively reduces over increasing number of desensitisations. As shown by Sloane et al., the percentage of patients with any breakthrough reaction during the DD procedures to chemotherapy and monoclonal antibodies decreased in time with a corresponding increase in the percentage of patients who tolerated the desensitization procedure during repeated cycles (Sloane et al., 2016). Patients initially presenting with anaphylaxis and desensitized in the intensive care setting proceeded to repeated successful desensitisations in the outpatient setting.

Secondly, although protocols are mostly empirical and the best and safest protocol is unknown, it has been reported that after two successful DD cycles, patients may tolerate even shorter subsequent protocols (Sloane et al., 2016).

Finally, DD is widely used in the management of immediate reactions, whereas in non immediate reactions where a T cell-mediated mechanism predominates the role of DD is still limited. However, in mild clinical conditions such as macula-papular exanthemas and fixed drug eruptions, some DD protocols are successfully applied (Castells et al., 2012; Scherer et al., 2013).

Overall, these observations raise the suspicion that other cells and mechanisms are involved in DD.

### Modulation of Adaptive Immune Response to the Drug During DD

The adaptive immune response sustained by drug-specific T cells and its modification during DD procedures has been scarcely evaluated until now*.* One study, focused on aspirin DD in patients suffering from aspirin-exacerbated respiratory disease (AERD), showed that one month after beginning of DD, no difference was detectable in the percentage of CD4^+^ T cells and their cytokine production (IL-2, IL-4 and IFN-g) in comparison with baseline (Atkas et al., 2013). However, the lack of effects on T cells described do not exclude long-term effects of DD.

Concerning other immediate hypersensitivity reactions (HRs), such as those induced by biological agents (BA), we have recently shown that drug-specific T cell proliferation to infliximab (IFX) was progressively reduced during DD procedures in a patient suffering from allergic asthma with grass sensitization who had experienced an IFX-induced anaphylaxis. Accordingly, the humoral response to the drug (anti-IFX antibodies titer) showed a parallel decrease over successful DD cycles (Vultaggio et al., 2020). These DD-induced modifications of both cellular and humoral response to IFX were drug-specific, as anti-grass pollen IgE remained positive during the entire protocol as well as the cellular response to Phl p5 was consistently positive in all tested samples over DD cycles.

Some data are emerging about the modifications of immune response toward biological agents (BA) during DD in patients with delayed reactions. Teraki and Shiohara have shown a decrease in the percentage of CD8+T cells infiltrating the lesion in allopurinol fixed drug eruptions during DD procedure (Teraki and Shiohara, 2004). Overall, these studies provide limited and controversial information, not allowing any significant understanding of the cellular immune mechanisms operating during DD.

### Drug Desensitization Increases Regulatory Cytokines

The impairment of effector responses observed during DD procedures suggested the involvement of regulatory mechanisms operating in successful DD, in a similar way to what happens during allergen immunotherapy. The effects of DD on regulatory cytokine levels in patients desensitized have been evaluated in both immediate and delayed drug hypersensitivity reactions. Gelincik and coworkers have described a significant increase in IL-10 serum levels 24 h within the end of DD procedures in 24 patients who underwent successful DD for several oral or parental culprit drugs. In the same case series, no changes of IL-4, IL-5 and IFN-γ levels were observed. The authors observed a greater increase in IL-10 levels in patients desensitized for chemotherapeutic drugs (Gelincik et al., 2019). An additional study focused on platinum desensitized patients for immediate HRs has confirmed the increase of IL-10 serum levels after DD, with a tendency to reach higher levels of IL-10 after multiple cycles (Tüzer et al. 2020). Regarding delayed HRs, in a case report about DD to allopurinol after a fixed drug eruption, an increase of IL-10 (and IL-6) production by peripheral blood mononuclear cells, was observed. Intracellular IL-10 contents in T cells, but not serum levels, have been analyzed in one study involving patients during desensitization to aspirin in AERD. A decrease of IL-10 (and IFN-γ) intracellular expression in CD4^+^ T cells, was observed after 1 month of desensitization (Aksu et al, 2014). This discrepancy may be caused by the type of reaction where DD has been applied (pathogenesis of AERD) and by the fact that serum levels were not evaluated in this study.

IL-10 is an important regulatory and anti-inflammatory cytokine, largely studied and involved in successful allergen immunotherapy (AIT) (Ni et al., 2015). Studies involving AIT showed the role for IL-10 production by T cells (Treg) and B cells (Breg) in inducing tolerance (Akdis et al., 2005, van de Veen, 2017). It important to note that before Treg and Breg cells appear, early desensitization effect of AIT seems to be associated with IL-10 produced by other cells, such as basophils and MCs (van de Veen et al., 2017). In fact, different cells may produce IL-10, such as cells of adaptive (T cells, B cells) and innate immunity (dendritic cells, natural killer T cells, eosinophils, neutrophils, basophils, MC) and keratinocytes (Saraiva and O’Garra et al., 2010). The cellular source of IL-10 production during DD has to be defined as yet, and further studies are required in this field, however we might speculate that the immunological effects of DD are similar to those observed during AIT.

IL-35 is the newest member of IL-12 family. It is a dimeric protein consisting of two separate subunits, an IL-12 subunit α chain (P35) and IL-27 subunit Epstein-Barr virus-induced gene 3 (EBI3) β chain; IL-35 has manifested suppressive actions on the immune system. It is secreted by a variety of cells, and then activates its receptors through JAK/STAT signaling to exert its anti-inflammatory and immunosuppressive effects (Zhang, et al., 2019). In a patient desensitized to IFX, serum IL-35 was highly increased after each DD cycle and a progressive increase of baseline values in serum samples collected before each cycle was observed (Vultaggio et al., 2020). Such response to high antigen dose during DD is likely comparable to that described for AIT in which IL-35 has been recently described to play a relevant role (Shamji and Durham, 2017; Shamji et al., 2018) and confirm the involvement of regulatory cytokines in the DD-related immunological mechanisms.

### Drug Desensitization Induces Drug-SpecificTreg Cells

Regulatory T (Treg) cells are a subset of CD4^+^ αβT cells that play a major role for maintaining self tolerance and preventing autoimmunity, limiting chronic inflammatory diseases, dampening homeostatic lymphocyte expansion, and suppressing immune responses to parasites and viruses and tumors, including that induced by therapeutic vaccines. The manipulation of Treg functions is an important goal of AIT, since a successful AIT is sustained by the generation of allergen-specific Tregcells (Palomares et al., 2010), and, as recently shown, in particular by T cells producing the regulatory cytokine IL-35 (called Tr35) (Shamji et al. 2018).

The involvement of Treg cells in DD mechanisms has been analyzed in few studies until now, mainly regarding DD after HRs to monoclonal antibodies. An increase of CD4^+^CD25 ^+^ cells and CD4^+^CD25 + FoxP3+ Treg cells in peripheral blood after DD for rituximab has been described in a successful procedure in a patient suffering from nephrotic syndrome (Aydogan et al., 2013). In addition, during DD for IFX, PBMC upon *in vitro* stimulation with IFX were able to produce IL-35 in a MHC-Class II-restricted manner, suggesting that the production of this regulatory cytokine is sustained by the presence of drug-specific Tr35. Notably these cells constitutively express check point molecules, including PD1 (Turnis et al., 2016), and accordingly, in the same case report, increased proportion of circulating CD3^+^CD4+PD1+ and CD3^+^CD4+Foxp3+ T cells were observed after the second and third cycle of desensitization. Regarding delayed reactions induced by allopurinol, Teraki and Shiohara showed that the number of CD4^+^CD25 ^+^ T cells increased in skin lesions after the beginning of DD, suggesting that Treg cells may migrate from blood to skin, where they might act to suppress effector T cells, that conversely decreased in proportion (from 91% to 35%) (Teraki and Shiohara, 2004). Even though these data obtained in single case reports must be considered preliminary, overall, they suggest a possible role of Tregulatory cells in the drug tolerance induced by DD.

## Discussion

Drug desensitization allows a transient clinical tolerance to the culprit drug by administering, in a short time, increasing amounts of the drug until reaching the therapeutic dose. Such procedure is to be applied mainly in reactive patients with no alternative treatment options.

Besides a profound change of MC reactivity with inhibition of their activation pathways and mediators’ release (only partially known until now), some reports strongly indicate that tolerance induced by DD implies the modulation of drug-specific response by regulatory mechanisms, confirming that this type of procedure deeply impacts on the immune response, more than that has been demonstrated to date. Of note, this regulatory activity is transient and lasts a short and variable period after stopping the treatment.

**Figure 1:**
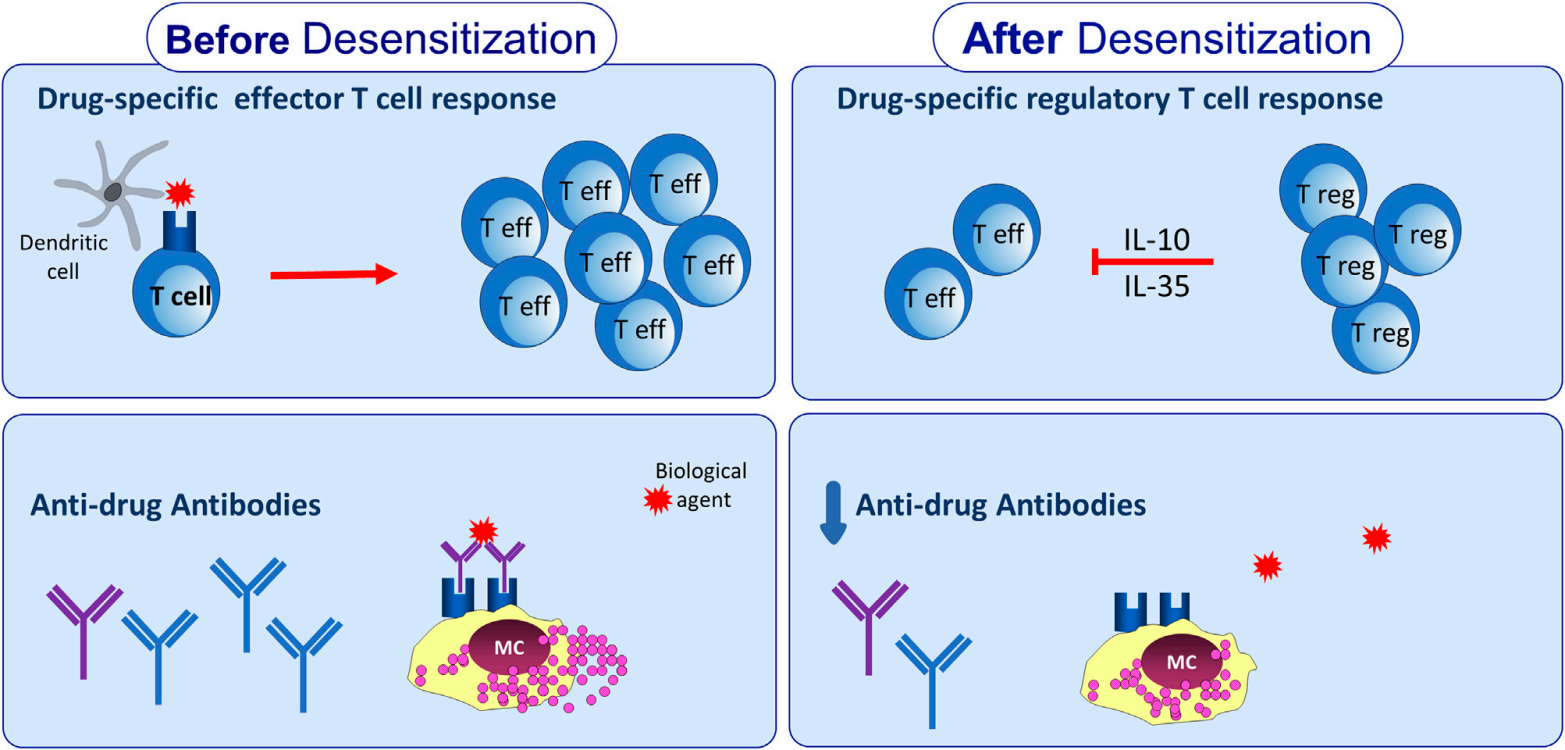
Adaptive immunological changes induced by drug desensitization. Drug desensitization may impact on adaptive immune response leading to reduction of anti-drug antibodies levels. In addition, drug-specific T cell response seems to be affected by drug desensitization, due to expansion of T regulatory cells able to produce IL-10 and IL-35.

Drug-specific immune response to BAs is down-regulated by a panel of regulatory cytokines, including the traditional IL-10 and a new molecule belonging to IL-12 family, IL-35. In particular, the activation/expansion of drug-specific Tr35 cells, occurring during the DD procedure to some BAs, may have a particular relevance in the mechanisms of DD tolerance, since IL-35 orchestrates other regulatory cells and cytokines. Figure 1 illustrates the immunological modifications of both umoral and cellular adaptive immune response to BA during DD.

It would be desirable to establish in the near future which regulatory cells (Treg, Breg, ILCreg, DCreg etc) are involved in each drug treatment, when they become operative during the DD procedure, and how long they last after stopping the DD. In addition, we cannot exclude that the type of drug, the route of administration, the dose and the scheme could influence the mechanisms operating in successful DD.

In conclusion, DD procedure induces two independent antigen-specific mechanisms: the release of controlled cellular mediators by MC over time and the development of the antigen-specific regulation of adaptive response. These mechanisms allow to safely and successfully reach the target dose of the drug.

## Author Contributions

AV and AM wrote the manuscript. FN designed the figure. EM, SB and EV revised the manuscript. All authors contributed to the article and approved the submitted version.

## Conflict of Interest

The authors declare that the research was conducted in the absence of any commercial or financial relationships that could be construed as a potential conflict of interest.
